# Cerebral Venous Sinus Thrombosis in a Child With Idiopathic Nephrotic Syndrome: A Case Report and Review of the Literature

**DOI:** 10.7759/cureus.11248

**Published:** 2020-10-29

**Authors:** Jiali He, Fang Yang

**Affiliations:** 1 Department of Pediatrics, The First Affiliated Hospital of Jinan University, Guangzhou, CHN

**Keywords:** child, nephrotic syndrome, cerebral venous sinus thrombosis

## Abstract

Cerebral venous sinus thrombosis is a rare and serious complication of nephrotic syndrome. A case of a five-year-old boy with nephrotic syndrome is described here. On the fourth day of admission, the child developed an occasional cough. A percutaneous renal biopsy was conducted to characterize the frequent-relapse nephrotic syndrome that was developed. After suspension of oral anticoagulants, the patient developed mild dizziness, headache, and vomiting. The child was diagnosed with intracranial venous sinus thrombosis based on data obtained using head computed tomography and magnetic resonance imaging. He recovered after receiving heparin and warfarin anticoagulants. We summarized the case and reviewed the literature here, showing that early diagnosis and treatment have a significant impact on the prognosis of this complication.

## Introduction

Cerebral venous sinus thrombosis (CVST) is a consequence of a combination of factors that cause abnormal cerebral venous hemodynamics. CVST as a complication in children with nephrotic syndrome (NS) is rarely reported. CVST is easily misdiagnosed or missed because of its atypical clinical manifestations. To improve prognosis, it is vital to diagnose this condition and treat it early. The purpose of the present article is to report a case of NS with CVST and to review existing cases in the literature.

## Case presentation

A five-year-old male child was diagnosed with NS six months prior to this admission. He was administered oral prednisolone and dipyridamole at doses of 2 mg/kg/d and 5 mg/kg/d, respectively, and went into remission within two weeks. Over the following six months, he relapsed twice. Half a day before this admission, he presented with proteinuria, no gross hematuria, no frequent urination pain, no swelling of eyelids and limbs, no cough and sputum, no dizziness, and no headache or fever. Before admission to the hospital, the dose of prednisolone administered was 2 mg/kg/d. On admission, the patient had a respiratory rate of 27 breaths/min, blood pressure of 117/82 mmHg, pulse rate of 117 beats/min, and weight of 22 kg. The results of the rest of the systemic examinations were normal.

**Figure 1:**
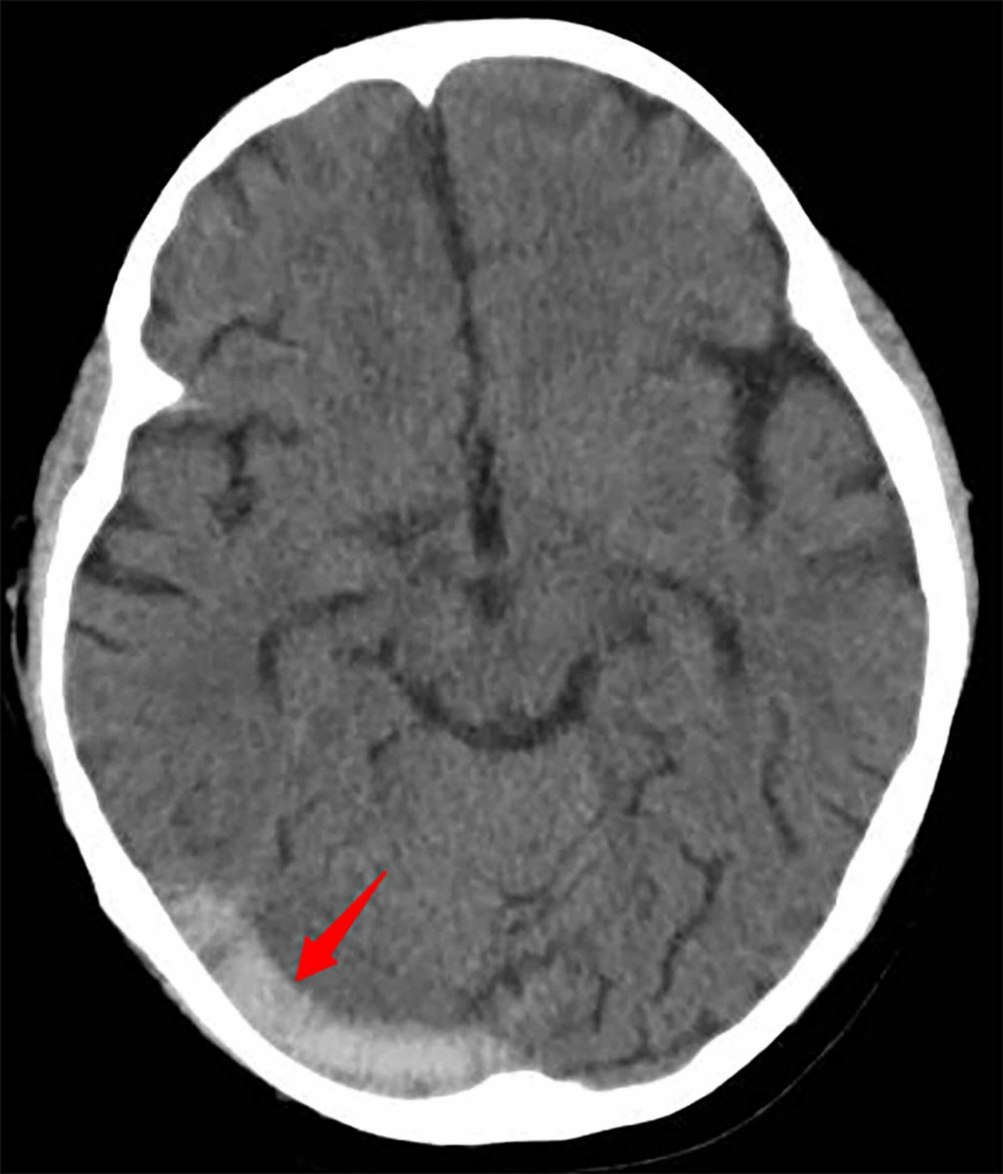
Brain CT On day five, brain CT shows the right sigmoid and the transverse sinuses with high density as shown by the red arrows.

**Figure 2:**
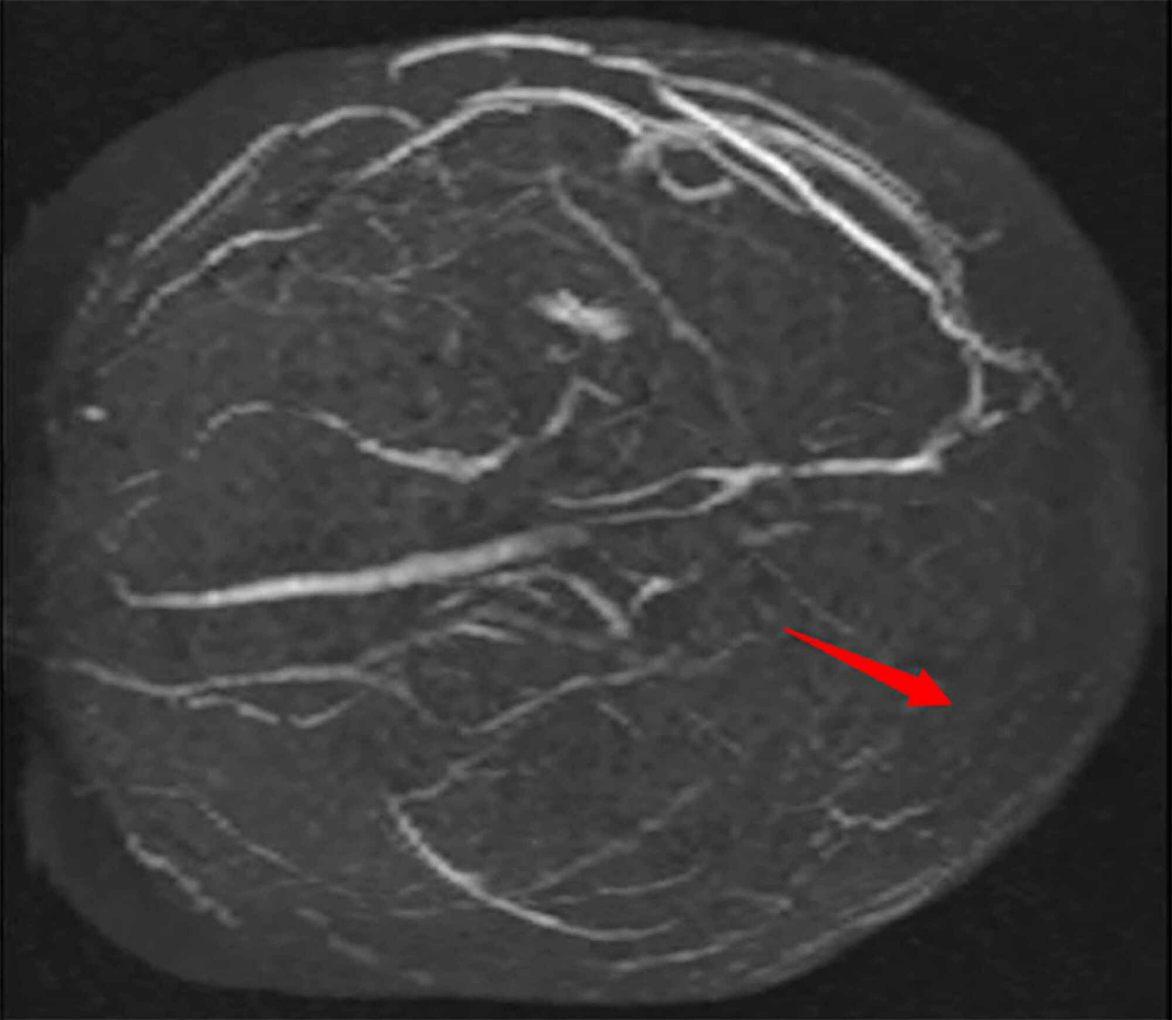
Brain MRV On day five, 3D MRV shows extensive sinovenous thrombosis, including the superior sagittal, right sigmoid, and transverse sinuses as indicated by the red arrows.

**Figure 3:**
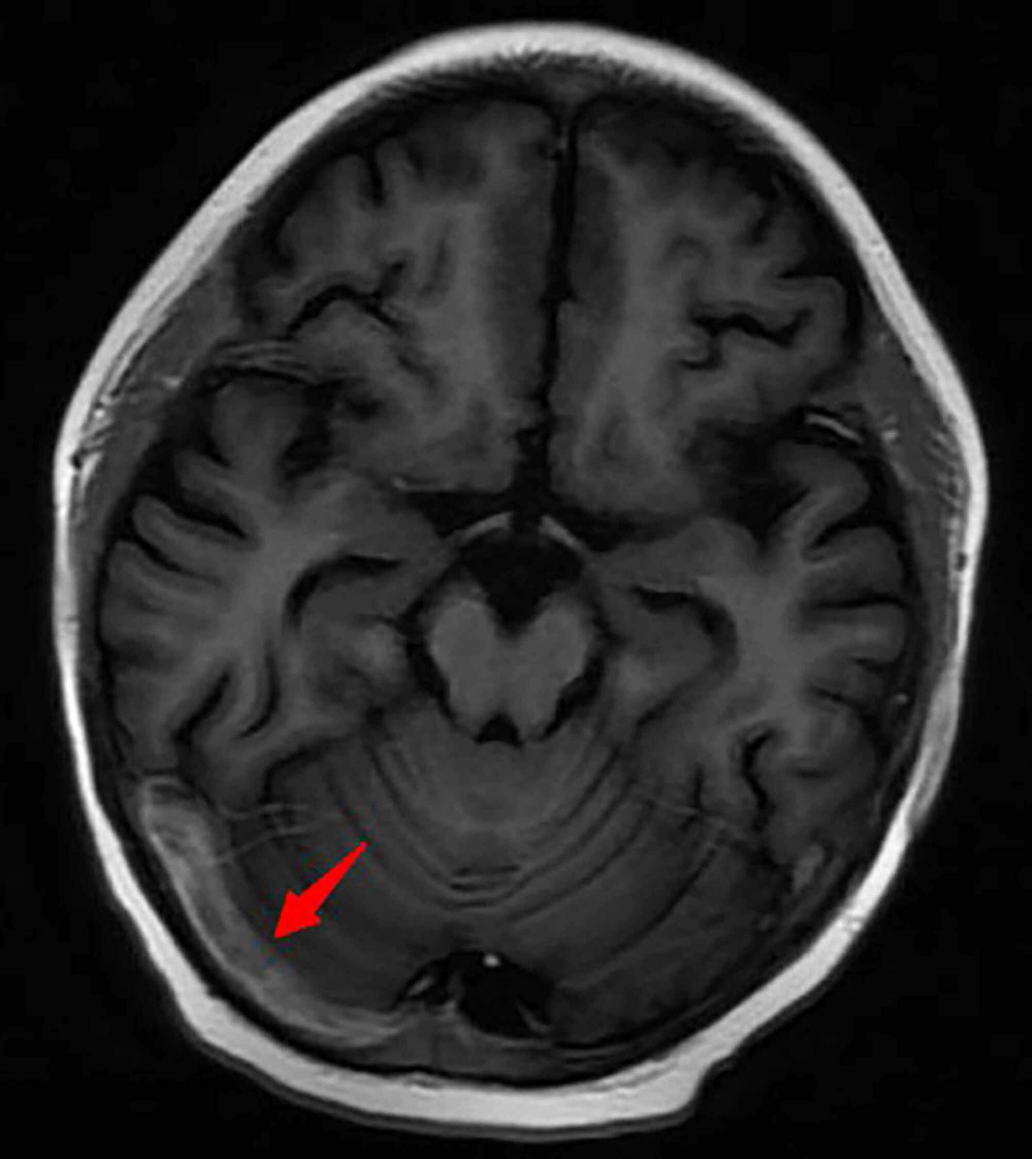
Brain MRI On day 17, 3D MRV shows that the thromboses are less severe than before.

**Figure 4:**
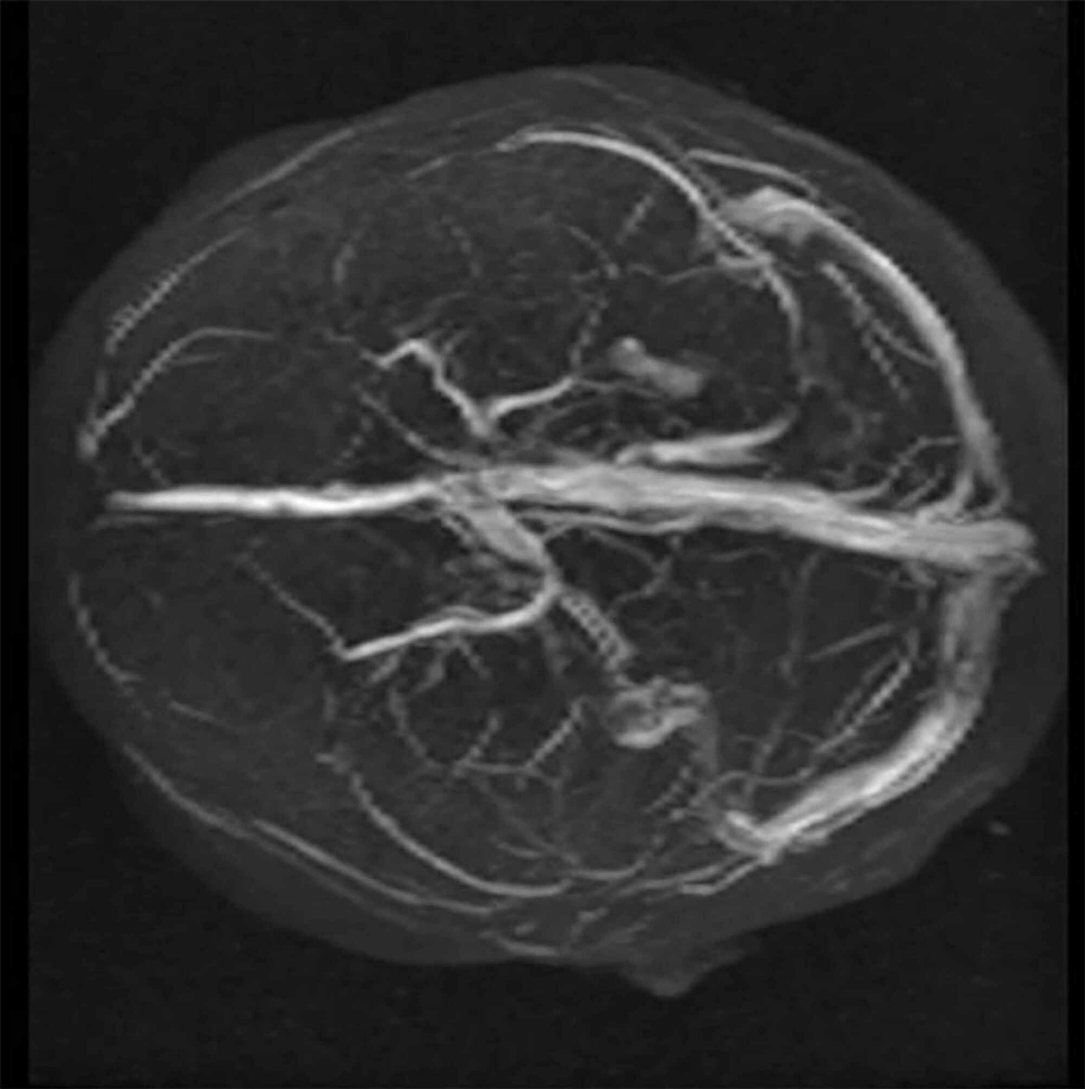
Brain MRV After three months, the thromboses had disappeared.

On day four, the patient began to cough. Percutaneous renal biopsy was performed to characterize NS. The day before the renal biopsy, dipyridamole and unfractionated heparin treatment was suspended. That night, the patient developed vomiting with intermittent headache. On the morning of the fifth day, his spirits deteriorated. His blood pressure was 115/72 mmHg. Drowsiness and no edema on face or lower extremities were observed. Bilateral pupils were equally large and round, each with a diameter of about 2.5 mm. They were sensitive to light reflection. Both pathological and meningeal irritation signs were negative. Other physical examinations were normal. The patient urgently underwent head computed tomography (CT) and magnetic resonance imaging (MRI). Laboratory examination showed heavy proteinuria of 220 mg/kg/d and serum albumin of 26.6 g/L. On day five, white blood cell count increased from 17.98×109/L to 21.89×109/L. Chest X-ray was normal. Brain CT and magnetic resonance venography (MRV) showed filling defects in the superior sagittal, right sigmoid, and transverse sinuses (Figures 1-2). On day 17, the thrombosis appeared to be less severe than on day five according to the MRV (Figure 3). After three months, the MRV results were normal (Figure 4).

The coagulation function at the onset of this hospitalization was changed compared with that on admission (Table 1).

**Table 1:** Dynamic alterations in coagulation function FDP fibrin degradation product; INR international normalized ratio

Days after admission	2	5	6	7	8
D dimer (ng/mL)	220	2680	4410	6390	4000
FDP (µg/mL)	4.0	7.25	14.96	25.79	16.32
Fibrinogen (g/L)	2.94	6.42	7.24	7.24	6.94
Prothrombin time (s)	12.1	12.6	12.3	14.1	15.2
INR	0.91	0.95	0.92	1.10	1.20

The initial diagnosis was frequently recurrent NS and the final diagnoses were CVST, frequently recurrent NS, acute upper respiratory infections, and nasosinusitis.

On day five, unfractionated heparin (125-200 IU/kg/d) was used and continued for 16 days. Dipyridamole was administered throughout hospitalization. Antithrombotic treatment (oral warfarin) was used throughout hospitalization and after the patient was discharged, with the target international normalized ratio (INR) between 2.0 and 2.5. After being discharged, the patient had an MRI review after two months. Three months after onset, results of head MRI were normal.

## Discussion

We searched databases using the following terms: “child” and “nephrotic syndrome” and “cerebral venous sinus thrombosis” or “cerebral sinovenous thrombosis.” The publication date was set between 1989 and 2019. Nine articles were retrieved (Table 2) [1-9].

**Table 2:** Similar studies retrieved from databases in this column, numbers refer to days after admission; NS, nephrotic syndrome; CVST, cerebral venous sinus thrombosis; M, male; F, female; SSS, superior sagittal sinus; ISS, inferior sagittal sinus; SS, straight sinus; LTS, left transverse sinus; RTS, right transverse sinus; LST, left sigmoid sinus; RST, right sigmoid sinus; CS, cavernous sinus; DSA, digital subtraction angiography; LMWH, low molecular weight heparin; rt-PA, recombinant tissue plasminogen activator; MRV, magnetic resonance venography

Author	Publication year	Journal	Cases	Age and gender	Type of NS	Clinical manifestation	CVST site	Imaging	Treatment
Rodrigues MM [1]	2003	Arq Neuropsiquiatr	1	9y; M	SSNS	headache, vomiting, upper abdominal pain	SSS, LTS	CT+MRI+MRV	heparin, warfarin
Gangakhedkar A [2]	2005	J Paediatr Child Health	1	9y; M	—	Vomiting, brief generalized tonic seizures	Sagittal sinus, SS	CT+MRI	heparin, LMWH, warfarin
Balci Y I [3]	2007	Eur J Pediatr	1	5y; M	SSNS	headache, vomiting	SSS, RTS	MRI	heparin, LMWH
Besbes LG [4]	2011	Case Rep Nephrol	1	7y; M	SDNS	headache, left eye strabismus	SSS, RTS, RST	CT+MR+MRV	heparin
Al-Rumayyan AR [5]	2014	Neurosciences (Riyadh)	1	10y; F	—	headache, vomiting, stomachache, fever, dehydration	SS	DSA+CT	rt-PA
Torres RA [6]	2014	Rev Bras Ter Int	1	2y; M	—	epileptic seizure, headache, vomiting, visual changes	SSS, LTS	MR	heparin
Kurt-Şükür ED [7]	2015	Nefrología	2	15y, 2y; 2M	SSNS in 2 cases	headache, vomiting	SSS, RST, TS	CT+MR	heparin
Kumar M [8]	2017	Sudan J Paediatr	1	5y; M	—	headache	SSS, ISS, TS, ST	MRV	heparin
Silva AI [9]	2018	J Bras Nefrol	1	5y; F	—	headache, vomiting	RTS	CT	heparin, warfarin

NS is a clinical syndrome that results from increased permeability of the glomerular filtration membrane to plasma protein. This leads to the loss of a large amount of plasma protein in the urine, which causes a series of pathophysiological changes. Clinical manifestations are proteinuria, hypoalbuminemia, edema, and hyperlipidemia. Its complications include infection, thrombus, acute renal injury, electrolyte and lipid metabolism disorders, adrenal crisis, and low T3 syndrome.

Common thrombosis complications in NS are deep vein and renal vein thrombosis and pulmonary embolism. These present as limb swelling, waist pain, fever, and gross hematuria. Factors leading to thrombosis in NS mainly include hypercoagulability, infection, and diuretic abuse. Hypercoagulability is caused by clot factor changes between coagulation and fibrinolytic systems, increased platelet count and function, and hyperlipidemia and steroid hormone use [10]. The infection caused by NS generally includes pneumonia, peritonitis, urinary tract infection, and bacteremia [11]. Moreover, this infection causes a decrease in blood volume, increase in blood viscosity, and loss of a large number of anticoagulation factors and proteins due to unreasonable application of diuretics.

CVST, which has an incidence rate of 0.67/100,000, is less frequent than other complications in pediatric NS [12]. The risk factors of CVST can be either infectious or non-infectious. Infectious factors include central nervous system infection [13]; facial, sinus, and oral infections; fungal and viral infections; and mastoiditis. The non-infectious factors include cerebral tumors [14], head traumas [15], internal jugular venous malformations, dural arteriovenous fistulas [16], blood system diseases (hemophilia, hereditary coagulation disorder, essential thrombocythemia, polycythemia vera, Evans syndrome), rheumatoid immune diseases (Behcet disease, antiphospholipid syndrome, systemic lupus erythematosus, and granulomatous vasculitis), endocrine diseases (diabetes and thyroid hyperfunction), paroxysmal nocturnal hemoglobinuria, dehydration, ulcerative colitis, drugs (cisplatin, methotrexate, oral contraceptives, and androgens), lumbar puncture, pregnancy, and puerperium [17]. Prethrombotic factors have also been reported to include protein C deficiency, factor V Leiden，protein S deficiency, antithrombin deficiency, and elevated factor VIII levels [18].

CVST also presents with various non-specific clinical features, such as headaches, dizziness, vomiting, decreased level of consciousness, twitching, and papilledema, which lead to CVST being misdiagnosed. This is especially true because children have difficulty expressing discomfort. The patient’s condition can progress rapidly within hours or days and can be life-threatening in severe cases. The diagnosis of choice is made using imaging, including head CT, MRI, MRV, and digital subtraction angiography (DSA). DSA, the gold standard for diagnosis, is invasive and expensive; therefore, it is seldom used. In general, head MRI and head MRV examinations can diagnose CVST. If the lesion area is not obvious and it is difficult to make a definitive diagnosis, DSA can be further performed. MRV has been recommended for CVST follow-up [19]. CVST treatment is mainly carried out using anticoagulant therapy. Anticoagulants include urokinase, low molecular weight heparin, and warfarin, which can be used in combination. The 2010 EFNS guidelines indicate that there is insufficient evidence to support the use of systemic or local thrombolysis in CVST patients. Additionally, the choice, dosage, route, or method of administration of the drug is not clear [20].

The review of the international literature [1-9] reveals that the majority of patients are male. Although their clinical manifestations are diverse, they mostly present with headache and vomiting. Regarding the thrombus site, the superior sagittal sinus is the most common. The treatment of choice is heparin anticoagulation followed by oral warfarin.

The patient in the present case took a large dose of glucocorticoid hormones for NS. He had no headaches or other symptoms at admission. There was frequent recurrence of the disease. Thus, a percutaneous renal biopsy was carried out to identify the pathological type. Oral dipyridamole was discontinued for one day prior to renal biopsy. The child developed a cough on the third day, and the head CT results showed sinusitis, indicating a respiratory infection. The next day, the patient suddenly presented with non-ejection-like vomiting and mild headache. Physical examination showed only mental fatigue; otherwise, the child was normal. The patient’s symptoms and signs were not obvious. Thus, misdiagnosis and delayed treatment were highly likely.

## Conclusions

CVST should be considered when there is a history of glucocorticoid hormone use at high doses, increased coagulation function, and infections. NS patients need to have their urinary protein levels controlled and serum albumin levels maintained to reduce the occurrence of a hypercoagulable state and thrombosis. These patients should also have their infections controlled in a timely manner. Infection will cause NS recurrence and increase the risk of CVST. 

In the present case, there was timely detection of headache symptoms. Thus, the patient was promptly diagnosed with NS complicated with CVST. After two weeks of heparin treatment and three months of warfarin treatment, the cranial MRI results showed significant improvement on day 13. The symptoms (such as headache), consciousness, physical examination, and cranial MRI results returned to normal after three months. The patient is still being followed up. 

In summary, when symptoms and clinical manifestations are non-specific, CVST should be considered in NS patients with infective inducement and consciousness disorders. Timely diagnosis and appropriate management can have a significant impact on the prognosis.
